# Role of deleterious single nucleotide variants in the coding regions of *TNFAIP3* for Japanese autoimmune hepatitis with cirrhosis

**DOI:** 10.1038/s41598-019-44524-5

**Published:** 2019-05-28

**Authors:** Takashi Higuchi, Shomi Oka, Hiroshi Furukawa, Minoru Nakamura, Atsumasa Komori, Seigo Abiru, Satoru Hashimoto, Masaaki Shimada, Kaname Yoshizawa, Hiroshi Kouno, Atsushi Naganuma, Keisuke Ario, Toshihiko Kaneyoshi, Haruhiro Yamashita, Hironao Takahashi, Fujio Makita, Hiroshi Yatsuhashi, Hiromasa Ohira, Kiyoshi Migita

**Affiliations:** 1 Molecular and Genetic Epidemiology Laboratory, Faculty of Medicine, University of Tsukuba, 1-1-1 Tennodai, 305-8575 Tsukuba, Japan; 2 Clinical Research Center, National Hospital Organization Nagasaki Medical Center, 2-1001-1 Kubara, 856-8562 Omura, Japan; 3 National Hospital Organization, Nagoya Medical Center, 4-1-1 Sannomaru, Naka-ku, 460-0001 Nagoya, Japan; 4 Department of Gastroenterology, National Hospital Organization, Shinshu Ueda Medical Center, 1-27-21 Midorigaoka, 386-8610 Ueda, Japan; 5 National Hospital Organization, Kure Medical Center, 3-1 Aoyama-cho, 737-0023 Kure, Japan; 6 National Hospital Organization, Takasaki General Medical Center, 36 Takamatsu-cho, 370-0829 Takasaki, Japan; 7 National Hospital Organization, Ureshino Medical Center, 2436 Shimojuku, 843-0393 Ureshino, Japan; 8 National Hospital Organization, Fukuyama Medical Center, 4-14-17 Okinogami-cho, 720-8520 Fukuyama, Japan; 9 National Hospital Organization, Okayama Medical Center, 1711-1 Tamasu, Kita-ku, 701-1192 Okayama, Japan; 10 National Hospital Organization, Higashinagoya National Hospital, 5-101 Umemorizaka, Meito-ku, 465-8620 Nagoya, Japan; 11 National Hospital Organization, Shibukawa Medical Center, 383 Shiroi, 377-0280 Shibukawa, Japan; 12 Department of Gastroenterology, Fukushima Medical University School of Medicine, 1 Hikarigaoka, 960-1295 Fukushima, Japan; 13 Department of Rheumatology, Fukushima Medical University School of Medicine, 1 Hikarigaoka, 960-1295 Fukushima, Japan

**Keywords:** Immunogenetics, Autoimmunity, Autoimmune hepatitis

## Abstract

Autoimmune hepatitis (AIH) is an autoimmune liver disease and cirrhosis is sometimes complicated with AIH at diagnosis, influencing its prognosis. *TNFAIP3* gene encodes A20, an inhibitor of nuclear factor-κB pathway, and is a susceptibility gene for autoimmune diseases. We investigated deleterious variants in the coding regions of *TNFAIP3* gene of Japanese AIH patients or those with cirrhosis. The deleterious variants in the coding regions of *TNFAIP3* gene were analyzed by the cycle sequencing method and the frequencies of deleterious *TNFAIP3* alleles of AIH or AIH with cirrhosis were compared with those of Japanese controls. The deleterious alleles in *TNFAIP3* were not associated with AIH. A significant association was shown for the deleterious alleles in *TNFAIP3* (*P* = 0.0180, odds ratio (OR) 4.28, 95% confidence interval (CI) 1.53–11.95) with AIH with cirrhosis at presentation. The serum IgM levels in AIH patients with deleterious alleles in *TNFAIP3* were tended to be lower than those without (*P* = 0.0152, *Q* = 0.1216). The frequency of deleterious alleles in *TNFAIP3* was higher in the AIH subset without the *DRB1* risk alleles than that with (*P* = 0.0052, OR 5.10, 95%CI 1.55–16.74). The deleterious alleles in *TNFAIP3*were associated with AIH with cirrhosis.

## Introduction

Autoimmune hepatitis (AIH) is an autoimmune liver disease with chronic progression^1^. Liver cirrhosis is sometimes complicated with AIH at diagnosis and influences its prognosis. AIH patients with cirrhosis had a worse survival^2^. The genetic and environmental factors are involved in the pathogenesis of AIH. A genome-wide association study (GWAS) on European population revealed that human leukocyte antigen (*HLA)* is the strongest genetic risk factor for AIH^3^. *HLA*-DRB1*03:01 and DRB1*04:01 were associated with AIH in European populations^4^. DRB1*04:01 and DRB1*04:05 were associated with AIH in Japanese populations^5–8^. DRB1*08:02 and DRB1*08:03 were also predisposing for the disease, when these alleles were possessed by individuals with DRB1*04:05^8^. The previous GWAS also suggested associations of single nucleotide variants in other genes outside of *HLA*^3^. Several candidate-gene approach studies reported weak genetic associations of single nucleotide variants with Japanese AIH in other genes than *HLA*; *STAT4*^9^, *PTPN22*^10^, *ICOS*^11^, *TNIP1*^12^, and *SH2B3*^13^.

*TNFAIP3* (tumor necrosis factor-α induced protein 3) gene encodes A20, an inhibitor of nuclear factor-κB (NF-κB) activation, and is a susceptibility gene for autoimmune diseases including systemic lupus erythematosus^14,15^ or rheumatoid arthritis^16,17^. A20 is a negative regulator of the NLRP3 inflammasome and myeloid cell specific deletion of A20 caused spontaneous arthritis in mice^18^. Analogically, inflammasome was activated in monocytes of non-transplanted AIH children and liver-transplanted children with *de novo* autoimmune hepatitis, but increased expression of A20 was observed in monocytes of liver-transplanted children without *de novo* autoimmune hepatitis^19^. Thus, A20 plays some important roles against autoimmune diseases. Recent studies reported that loss of function (nonsense or frameshift variants) or deleterious missense variants (variants that changed amino acid residues on positions highly conserved across orthologs) in *TNFAIP3* dominantly caused an autoinflammatory disease with Behçet’s disease-like symptoms, the haploinsufficiency of A20 syndrome (HA20)^20–22^. However, the symptoms of HA20 also included those of autoimmune diseases, arthritis, nephritis, vasculitis, or hepatitis^23^. Thus, we investigated the variants in the coding regions of *TNFAIP3* gene of Japanese AIH patients by the cycle sequencing method and tried to compare the frequencies of deleterious *TNFAIP3* alleles of AIH or AIH with cirrhosis with those of Japanese controls.

## Results

### Associations of deleterious alleles in *TNFAIP3* with AIH with cirrhosis

The exons and its boundaries of *TNFAIP3* gene were amplified by 9 primer sets to detect variants by the direct sequencing method. Seven single nucleotide variants were found in the coding regions of *TNFAIP* in 360 AIH patients. No variant was detected in the splice sites of *TNFAIP3* gene and no insertion or deletion was found. Two (rs200595071 and rs769014911) of these variants were synonymous and the other five were non-synonymous. Of these five non-synonymous variants, three variants [c.116A > G (p.H39R, chr6:137871343 in GRCh38), c.305A > G (rs146534657, p.N102S, chr6:137874854), and c.1897G > C (p.E633Q, chr6:137879342)] were predicted to be deleterious (probably damaging, affect protein function, disease causing, or deleterious) by all five protein prediction algorithms, and two [c.380T > G (rs2230926, p.F127C, chr6:137874929) and c.2140C > T (rs369155845, p.P714S, chr6:137881086)] were predicted to be neutral (benign, tolerated, polymorphism, or neutral) by all. Thirteen alleles of these three deleterious variants (one c.116A > G, eleven c.305A > G, and one c.1897G > C, Fig. S1) were detected in twelve AIH patients and one was estimated to be a compound heterozygote for c.116A > G and c.305A > G.

Deleterious allele frequencies in the AIH patients and the Japanese controls are shown in Table 1. No significant association with AIH was detected for the deleterious alleles in *TNFAIP3*. When AIH patients with cirrhosis at presentation were compared with the Japanese controls, a significant association was shown for the deleterious alleles in *TNFAIP3* (*P* = 0.0180, odds ratio (OR) 4.28, 95% confidence interval (CI) 1.53–11.95, Table 1).

**Table 1:** Association of a burden of deleterious variants in *TNFAIP3* with the risk for AIH or AIH with cirrhosis.

	2n		Deleterious allele number	*P*	OR	95%CI
AIH	720		13 (1.81)	0.2323	1.42	(0.79–2.54)
AIH with cirrhosis at presentation	76		4 (5.26)	0.0180	4.28	(1.53–11.95)
Control	7094		91 (1.28)			

AIH: autoimmune hepatitis, OR: odds ratio, CI: confidence interval. Allele frequencies are shown in parenthesis (%). Deleterious allele frequencies of AIH or AIH with cirrhosis were compared with those of Japanese controls by Fisher’s exact test using 2 × 2 contingency tables under the allele model.

### Clinical features of the AIH patients with or without deleterious alleles in *TNFAIP3*

The demographic features of AIH patients with or without deleterious alleles in *TNFAIP3* were analyzed (Table 2). The serum levels of IgM in AIH patients with deleterious alleles in *TNFAIP3* were tended to be lower than those without (*P* = 0.0152, *Q* = 0.1216).

**Table 2:** Comparison of the demographics between AIH patients with or without deleterious variants in *TNFAIP3*.

	Deleterious variant (+) AIH	Deleterious variant (−) AIH	*P*	FDR *Q*
Number	12	348		
Male, n (%)	3 (25.0)	40 (11.5)	*0.1621	0.4183
Age at onset, years (SD)	54.2 (15.3)	59.3 (13.4)	0.2615	0.4183
Mean age, years (SD)	56.0 (16.7)	63.1 (13.4)	0.1196	0.4183
T-Bil (mg/dl) (SD)	4.3 (4.0)	3.7 (5.0)	0.2491	0.4183
AST (IU/L) (SD)	470.3 (443.3)	469.6 (549.9)	0.9281	0.9281
ALT (IU/L) (SD)	647.4 (691.6)	502.7 (503.3)	0.5524	0.7365
IgG (mg/dl) (SD)	2535.8 (934.7)	2409.3 (898.0)	0.7107	0.8122
IgM (mg/dl) (SD)	110.3 (59.0)	209.3 (231.0)	0.0152	0.1216

AIH: autoimmune hepatitis, Deleterious variant (+) AIH: AIH patients with deleterious variants in *TNFAIP3*, Deleterious variant (−) AIH: AIH patients without deleterious variants in *TNFAIP3*, T-Bil: total bilirubin, AST: aspartate aminotransferase, ALT: alanine aminotransferase, IgG: immunoglobulin G, IgM: immunoglobulin M. Numbers or average values of each group are shown. Percentages or standard deviations are shown in parenthesis. Association was tested between AIH patients with or without deleterious single nucleotide variants by Fisher’s exact test using 2 × 2 contingency tables or Mann-Whitney’s U Test. *Fisher’s exact test was employed. To correct for multiple testing, the false discovery rate (FDR) *Q*-value was calculated.

### Associations of deleterious alleles in *TNFAIP3* with AIH subsets with or without the *DRB1* risk alleles

The gene-gene interaction between *TNFAIP3* and *DRB1* was also investigated (Table 3). The frequency of deleterious alleles in *TNFAIP3* was higher in the AIH subset without the *DRB1* risk alleles than that with (*P* = 0.0052, OR 5.10, 95%CI 1.55–16.74).

**Table 3:** Deleterious allele frequencies of *TNFAIP3* in the AIH patients with or without the *DRB1* risk alleles.

	2n	Deleterious allele number	*P*	OR	95%CI
AIH without the *DRB1* risk alleles	226	9 (3.98)	0.0052	5.10	(1.55–16.74)
AIH with the *DRB1* risk alleles	496	4 (0.81)			

AIH: autoimmune hepatitis, OR: odds ratio, CI: confidence interval. Allele frequencies are shown in parenthesis (%). Association was tested between AIH patients with or without the *DRB1* risk alleles by Fisher’s exact test using 2 × 2 contingency tables under the allele model. The *DRB1* risk alleles were DRB1*04:01, *04:05, *08:02, or *08:03.

## Discussion

The present study revealed that deleterious variants in *TNFAIP3* were predisposing for AIH with cirrhosis in a Japanese population. *TNFAIP3* encodes A20, an inhibitor of the NF-κB signaling pathway, and is a susceptibility gene for autoimmune diseases and HA20^14–17,20–23^. A20 is a negative regulator of the NLRP3 inflammasome and plays some important roles against autoimmune diseases^18,19^. *TNIP1* is a predisposing gene in AIH^12^, and encodes an adaptor protein binding to A20. These data suggested the common signaling pathways in the pathogenesis of AIH, other autoimmune diseases, and HA20.

The progression pattern of AIH patients with cirrhosis at presentation would be smoldering and latent and the prognosis of AIH with cirrhosis was worse^2^. Cirrhosis at presentation was also a risk factor of development of hepatocellular carcinoma^24^. However, an age at onset was older in AIH patients with cirrhosis at presentation^2^, but that of AIH patients with deleterious variants in *TNFAIP3* was not older (Table 2), suggesting the existence of differential subsets of AIH. Deleterious variants in *TNFAIP3* were revealed to be risk factors for AIH with cirrhosis (Table 1). Thus, deleterious variants in *TNFAIP3* would modulate the progression pattern of AIH, contribute to configure the subset of AIH, and influence the prognosis of AIH. Further observation studies were needed to clarify the prognosis and the progression pattern of AIH patients with deleterious variants in *TNFAIP3*. In the present study, three deleterious variants in *TNFAIP3* (c.116A > G, c.305A > G, and c.1897G > C) were detected in Japanese AIH patients, but none of them were reported to cause HA20. One of the deleterious variants in *TNFAIP3* (c.305A > G) was appeared to be related to the poor clinical outcome of rheumatoid arthritis^16^, suggesting that deleterious variants in *TNFAIP3* are modulating symptoms of autoimmune diseases.

The association of deleterious variants in *TNFAIP3* was also analyzed between patients with or without the *DRB1* risk alleles. The frequency of deleterious variants in *TNFAIP3* was higher in the AIH patients without the *DRB1* risk alleles than those with. These data suggested that deleterious variants might not predispose AIH in individuals with the strongest genetic risk factor, the *DRB1* risk alleles. It was also possible that deleterious variants would be readily found in the individuals without the *DRB1* risk alleles. Serum IgM levels in AIH with deleterious variants in *TNFAIP3* were tended to be lower than those without (Table 2). Since AIH patients with higher serum IgM levels were suspected to be overlapped with primary biliary cholangitis, the AIH patients with lower serum IgM levels would be patients with lower probability of the overlap. It was reported that IgM levels of AIH patients with DRB1*04:05, one of the *DRB1* risk alleles, were higher than those without^6,8^, explaining the decreased IgM levels in AIH patients with deleterious variants. Since *DRB1* and *TNFAIP3* were distantly located on the different arms of chromosome 6 (*DRB1*: 6p21.32, 32578769–32589848 in GRCh38, *TNFAIP3*: 6q23.3, 137867188–137883312), linkage disequilibrium was not strong between these two loci (*r*^*2*^ = 0.003, between the *DRB1* risk alleles and deleterious variants in *TNFAIP3* in AIH patients), suggesting that deleterious variants in *TNFAIP3* and the *DRB1* risk alleles would be independently associated with AIH. However, the independence could not be confirmed by logistic regression analysis, because the genotypes of the Japanese controls were not available.

To the best of our knowledge, this is the first report of the association of deleterious variants in *TNFAIP3* with Japanese AIH with cirrhosis. Since the sample size of this study was limited and the frequencies of deleterious variants were low, the association was modest. The associations should be confirmed in future large scale studies.

## Materials and Methods

### Patients and controls

A total of 360 AIH patients were recruited from National Hospital Organization (NHO) hospitals. The clinical information of the enrolled patients was obtained and 38 had cirrhosis at presentation^2,24^. All the AIH patients were native Japanese living in Japan and satisfied the criteria of International Autoimmune Hepatitis Group for diagnosis of AIH^25^. The allele frequencies of single nucleotide variants in *TNFAIP3* gene in Japanese population were referred to 3.5KJPN panel from the genome cohort study of Tohoku Medical Megabank Organization (https://ijgvd.megabank.tohoku.ac.jp/)^26–28^. The distribution pattern of the age and gender of the controls was described elsewhere (https://ijgvd.megabank.tohoku.ac.jp/statistics/statistics-3-5kjpn-all/). This study was reviewed and approved by NHO Central IRB and University of Tsukuba Research Ethics Committee. Written informed consent was obtained from each individual. This study was conducted in accordance with the principles expressed in the Declaration of Helsinki.

### Genotyping

Genotyping of *TNFAIP3* gene was performed by the direct sequencing method of PCR products amplified with previously reported primers^16^ for exon 2 to exon 9 and BigDye Terminator v3.1 Cycle Sequencing Kit (Thermo Fisher Scientific Inc., Waltham, MA) using Applied Biosystems 3130xl Genetic Analyzer (Thermo Fisher Scientific Inc.). Deleterious alleles were defined by missense variants annotated as deleterious by all five protein prediction algorithms of PolyPhen-2 HumDiv (http://genetics.bwh.harvard.edu/pph2/index.shtml, probably damaging), PolyPhen-2 HumVar (http://genetics.bwh.harvard.edu/pph2/index.shtml, probably damaging), SIFT (http://sift.bii.a-star.edu.sg/, affect protein function), Mutation Taster (http://www.mutationtaster.org/, disease causing), and Likelihood Ratio Test Predictions (http://www.genetics.wustl.edu/jflab/lrt_query.html, deleterious)^29–34^. *DRB1* genotyping results of the AIH patients were reported previously^7,8^. DRB1*04:01, *04:05, *08:02, or *08:03 were designated as the *DRB1* risk alleles for AIH^8^.

### Statistical analysis

The distribution of deleterious allele frequencies in AIH patients or AIH patients with cirrhosis was compared with those in Japanese controls by Fisher’s exact test using 2 × 2 contingency tables under the allele model^35^. The clinical phenotypes of AIH patients with deleterious alleles were compared with those without by Fisher’s exact test using 2 × 2 contingency tables or Mann-Whitney’s U Test. The distribution of deleterious allele frequencies was compared between AIH patients with or without the *DRB1* risk alleles by Fisher’s exact test using 2 × 2 contingency tables under the allele model. Correction for multiple testing was performed by calculating false discovery rate *Q*-value^36^.

## Supplementary information

Supplementary Figure S1

## Data Availability

All relevant data are within the manuscript and its Supplementary Information files.
