# Supplementary material for: Role of deleterious single nucleotide variants in the coding regions of TNFAIP3 for Japanese autoimmune hepatitis with cirrhosis
Source: Sci Rep. 2019 May 28;9:7925. doi: 10.1038/s41598-019-44524-5 (PMC6538649; doi:10.1038/s41598-019-44524-5)
Supplement: Supplementary file 1 — Supplementary Figure S1 [file 41598_2019_44524_MOESM1_ESM.pdf]

Supplementary information

Role of deleterious single nucleotide variants in the coding regions of *TNFAIP3* for Japanese autoimmune hepatitis with cirrhosis.

Takashi Higuchi[1], Shomi Oka[1], Hiroshi Furukawa[1], Minoru Nakamura[2], Atsumasa Komori[2], Seigo Abiru[2], Satoru Hashimoto[2], Masaaki Shimada[3], Kaname Yoshizawa[4], Hiroshi Kouno[5], Atsushi Naganuma[6], Keisuke Ario[7], Toshihiko Kaneyoshi[8], Haruhiro Yamashita[9], Hironao Takahashi[10], Fujio Makita[11], Hiroshi Yatsuhashi[2], Hiromasa Ohira[12], and Kiyoshi Migita[2,13].

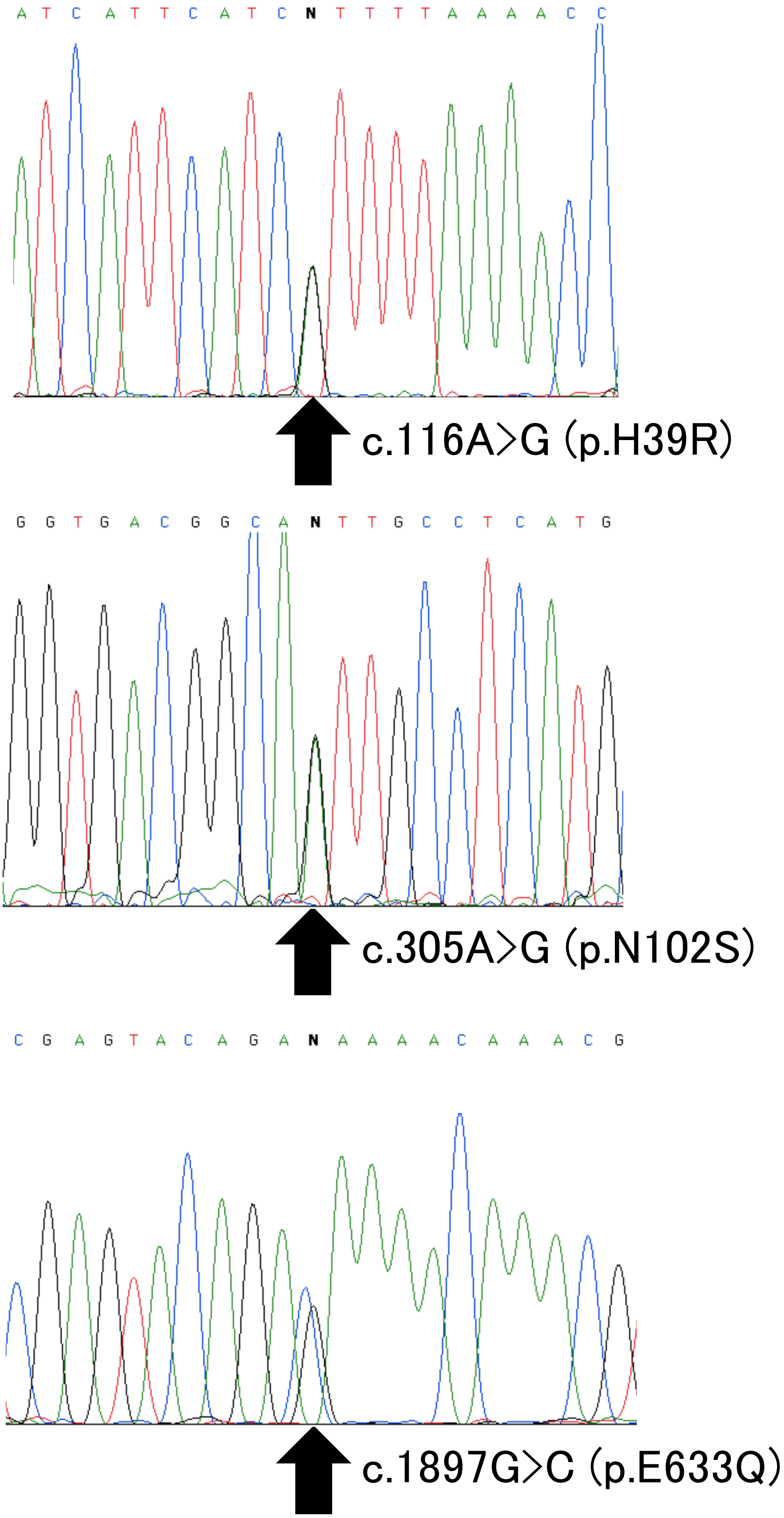

Supplementary Figure S1. Chromatograms of the cycle sequencing for the deleterious variants in *TNFAIP3* gene. Heterozygous variants were indicated by the arrows.
